# Relationships between C_3_ Plant Foliar Carbon Isotope Composition and Element Contents of Grassland Species at High Altitudes on the Qinghai-Tibet Plateau, China

**DOI:** 10.1371/journal.pone.0060794

**Published:** 2013-04-02

**Authors:** Yong-Chun Zhou, Jiang-Wen Fan, Warwick Harris, Hua-Ping Zhong, Wen-Yan Zhang, Xi-Lei Cheng

**Affiliations:** 1 Institute of Geographic Sciences and Natural Resources Research, Chinese Academy of Sciences, Beijing, China; 2 Liaoning Academy of Environmental Sciences, Shenyang, Liaoning Province, China; 3 Landcare Research, NZ Ltd, Lincoln, Canterbury, New Zealand; DOE Pacific Northwest National Laboratory, United States of America

## Abstract

Relationships of foliar carbon isotope composition (δ^13^C) with foliar C, N, P, K, Ca, Mg contents and their ratios of 219 C_3_ species leaf samples, obtained in August in 2004 to 2007 from 82 high altitude grassland sites on the Qinghai-Tibet Plateau China, were examined. This was done with reference to the proposition that foliar δ^13^C increases with altitude and separately for the life-form groups of graminoids, forbs and shrubs and for the genera *Stipa* and *Kobresia*. For all samples, foliar δ^13^C was negatively related to foliar K, P and ∑_K+ Ca+ Mg_, and positively correlated to foliar C, C/N and C/P. The significance of these correlations differed for the taxonomic and life-form groups. Lack of a relationship of foliar δ^13^C with foliar N was inconsistent with the majority of studies that have shown foliar δ^13^C to be positively related to foliar N due to a decrease of C_i_/C_a_ (the ratio between intercellular and atmospheric concentration of CO_2_) and explained as a result of greater photosynthetic capacity at higher foliar N concentration. However this inconsistency relates to other high altitude studies that have found that photosynthetic capacity remains constant as foliar N increases. After accounting for the altitudinal relationship with foliar δ^13^C, of the elements only the K effect was significant and was most strongly expressed for *Kobresia*. It is concluded that factors critical to plant survival and growth at very high altitudes, such as low atmospheric pressure and low temperatures, may preclude expression of relationships between foliar δ^13^C and foliar elements that have been observed at lower altitudes.

## Introduction

In the last 20 years, determination of foliar carbon isotope composition (δ^13^C) has been a powerful tool in plant ecophysiological studies [1]. Foliar δ^13^C is related to the C_i_/C_a_ ratio (the ratio between intercellular and atmospheric concentration of CO_2_) [2], and C_i_/C_a_ is determined by the balance between stomatal conductance (g_s_) and photosynthetic capacity (A) [3], so environmental factors may cause change of δ^13^C through their effect on A or g_s_. Thus, much effort has been directed towards investigating the relationships between δ^13^C and environmental factors [4]–[8].

Compared with a mass of systemic research on correlations of δ^13^C with various environmental factors, fewer studies have been made of relationships between δ^13^C and biotic factors, such as leaf nutrient concentration. Nevertheless, some leaf nutrients are related to g_s_ or A, so they may be also related to δ^13^C. For example, nitrogen (N) is an essential nutrient that has important roles related to plant growth, and its positive correlation with photosynthetic rates and chlorophyll pigment content have been reported [9]–[14]. Phosphorus (P) is associated with photosynthesis indirectly through the effects of orthophosphate on Calvin cycle enzymes [15]. Other nutrients, e.g., potassium (K), calcium (Ca), and magnesium (Mg), influence photosynthetic capacity and/or stomatal conductance in several ways, e.g., K is found to play a crucial role in regulation of stomatal function, osmoregulation, enzyme activity and cell expansion [16]–[18]. Ca is important in the regulation of water loss and stomatal closure [19], [20]. Mg, which occupies the center of the porphyrin nucleus of the chlorophyll molecule, has an important function in activating enzymes related to the synthesis of protein, RNA and DNA [21]. In addition, a negative correlation between ash content and δ^13^C has been widely observed [22]–[27]. This is because minerals are mostly passively transported in plant organs via the xylem flux and accumulated in place, so a plant that needs more water for the same biomass accumulation (lower δ^13^C) would be proportionally richer in minerals (higher ash content). Conversely, carbon (C) content should be positively correlated to δ^13^C [28], because if the leaf ash content is high, the leaf C content is low.

Previous investigations of relationships between nutrient elements and δ^13^C have mostly focused on K and N [22], [28]–[33]. This is because leaf K concentration has been proposed as a surrogate of δ^13^C and N is the closest positive relative to photosynthesis [30], [34], [35]. In general, foliar δ^13^C is negatively related to foliar K and positively related to foliar N [28], [29], [32], [33]. These results were predicated on the basis that K is mainly passively transported in plant organs via the xylem flux and that plants with high foliar N content have high photosynthetic capacity. However, as K plays key roles in regulating stomatal movement, higher foliar K concentration could cause higher stomatal sensitivity to water stress and lower stomatal conductance (higher δ^13^C), and some research has found positive relationship between foliar K and foliar δ^13^C [22], [31]. In addition, Cordell et al. [36] found that photosynthetic capacity remains constant with increases of foliar N at high altitudes, suggesting that foliar δ^13^C is not positively related to foliar N in these areas.

Investigating the relationships between foliar δ^13^C and C/N, C/P and N/P for various species and conditions, especially in extreme environments, such as very high altitudes, could have important implications for ecosystem analysis, because it links plant C/N/P stoichiometry with plant transpiration, and therefore integrates carbon, nutrient and hydrological cycles. Cernusak et al. [37] observed a positive correlation between leaf N/P ratio and WUE (δ^13^C). However, the species and environmental conditions involved in that study were limited and further testing of the relationship between the leaf N/P ratio and WUE is needed for more species and environmental conditions.

There have been few studies on nutrient elements-foliar δ^13^C relationships conducted in the very extensive high altitude environment of the Qinghai-Tibet Plateau, China. Moreover, research on nutrient elements-δ^13^C relationships has mostly involved trees [28], [32], [38] and crops [23], [26], [39], and has seldom involved species of natural grasslands [35]. Recent studies have indicated the dominating influence of altitude related factors on foliar δ^13^C, and emphasized atmospheric pressure related effects rather than those of temperature and water availability effects [7], [8]. Therefore it is important to account for altitude related effects when investigating relationships between foliar elements and foliar δ^13^C.

The objectives of this study for specified taxonomic plant groups were (1) to investigate relationships between foliar δ^13^C and foliar C, N, P, K, Ca and Mg, (2) to investigate relationships between foliar δ^13^C and nutrient ratios, i.e., C/N, C/P and N/P, as these nutrient ratios are also important plant ecophysiological measures which can provide important information about nutrient limitations to primary productivity [37], [40], (3) to consider whether relationships that have been previously indicated between foliar δ^13^C and leaf elements are subsidiary or coincidental to the reduction of discrimination against ^13^C fixation by plants that occurs with increased altitude, and (4) to specifically examine whether the relationships between foliar δ^13^C and foliar N and K for plants growing in high altitude environments are different from the general relationships previously shown between these characters.

## Materials and Methods

### Study area

**Figure 1:**
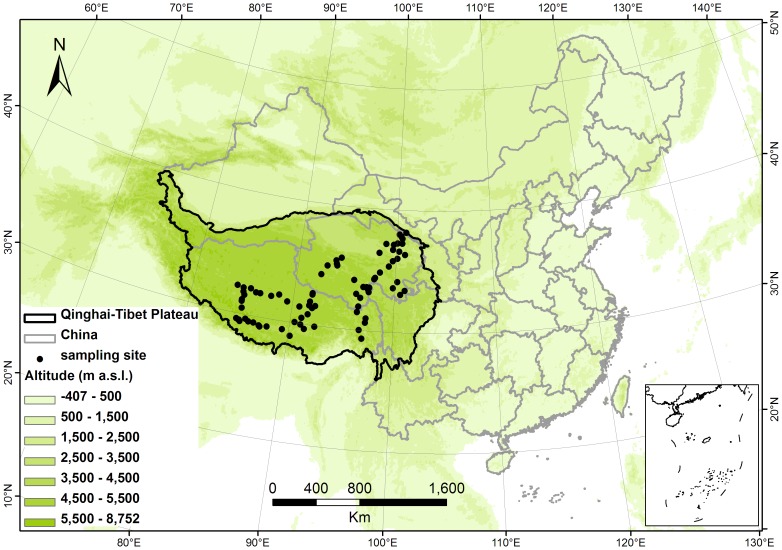
Locations of the 82 sampling sites on the Qinghai-Tibetan Plateau, China.

Plant samples were obtained from natural grasslands at 82 sites on the Qinghai-Tibet Plateau, southwest China (Figure 1) within an area of about 1400×650 km^2^, extending for ∼20° of longitude from 84.34° to 101.01° E, ∼10° latitude from 28.89° to 38.18° N, and altitudes from 2954 to 5269 m a.s.l. Atmospheric pressures in the altitude range vary from 71.1 to 53.3 kpa, equivalent to 70.2% and 52.6% of the atmospheric pressure at sea level. According to Chinese law, ownership of the grasslands of China belongs to all the people of China. Consequently herdsmen have grassland-use rights but not ownership. Our field work was permitted and assisted by local government agencies particularly the Animal Husbandry Bureau and Grassland Management Station of Qinghai and Tibet provinces. We also obtained the permission and help of local herdsmen.

The Qinghai-Tibet Plateau is the highest plateau in the world with an average elevation of about 4500 m. It is a critically important catchment including the headwaters of the Yangtze, Yellow (Huang He) and Lantsang (Mekong) rivers. The Plateau's climate is warm and humid in summer and cold and dry in winter. Mean annual precipitation (MAP) ranges from 100 mm to 800 mm, and mean annual temperature (MAT) is mostly in the range from −5.8 to 3.7°C but extends up to 12°C. Relationships between foliar δ^13^C and MAP and MAT in the region were considered in a previous study and found to be secondary to relationships with altitude related atmospheric pressure [7]. In this paper the focus is on relationships between foliar δ^13^C and leaf element contents.

Alpine meadow and alpine steppe are the main grassland types and the sedges *Kobresia pygmaea*, *Kobresia tibetica*, and *Kobresia humilis*, the grass *Stipa purpurea*, and the shrub *Dassiphora fruticosa* are typical dominant species. Soils are simple zonal types with alpine cold desert soil, alpine steppe soil and meadow soil the most widespread. There are also scattered areas of saline soil, alkali soil, sandy soil, takyr and other soils.

### Sampling

Samples were obtained from actively growing plants in August in 2004 to 2007. The plant species sampled were common, dominant and widespread. To minimize influences of complicating factors (such as grazing) on measurements, the sites sampled were representative of the natural grassland communities of the region and were protected by fences, not grazed, and not close to human habitation. The sites were separated from each other by about 50 km and the geographic position of each was recorded using a Magellan GPS (Garmin, Kansas, USA). At each site, samples were obtained from the dominant species, with five to 20 individual plants randomly sampled for each species. Each sampled species at each site was bulked together in an envelope as one sample and transported for laboratory analysis. A total of 219 C_3_ species leaf samples representing 55 genera in 19 families were obtained from the 82 sites.

### Leaf nutrient element concentration and stable carbon isotope analysis

Leaf nutrient element concentration and stable carbon isotope determinations were made for the 219 species leaf samples at the central physicochemical laboratory of the Institute of Geographic Sciences and Natural Resources Research, Chinese Academy of Sciences, Beijing. Depending on leaf size, three to 20 leaves (mostly five) from at least three different adult individual plants were selected for each species sample for each site. Leaves were washed with distilled water and air-dried, then oven-dried at 80°C for 48 h, before grinding to fine powder for analysis. ^13^C/^12^C ratio and total concentrations of nitrogen (N) and organic carbon (C) were determined by an isotope mass spectrometer (Thermo, MAT-253). Carbon isotope composition δ^13^C (‰) in leaf samples was calculated as:



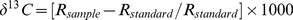
(1)


Where R_sample_ and R_standard_ respectively are the ^13^C/^12^C ratio in the leaf and the standard. The universally accepted standard of Pee Dee Belemnite (PDB) was used. The precision of isotope composition measurement was 0.1‰.

Leaf powder was digested by concentrated nitric acid and perchloric acid and then extracted solutions were determined for total concentrations of P, K, Ca and Mg on an Inductively Coupled Plasma Optical Emission Spectrometer (PerkinElmer, Optima 5300DV).

### Data analysis

Data analyses were conducted using SPSS (ver.18.0; SPSS Inc., USA). Simple correlation and regression analysis was applied to investigate relationships between foliar nutrient concentrations and both altitude and foliar δ^13^C using the values for the 219 species leaf samples and also for data extracted for the genera *Kobresia* and *Stipa*. To explore whether these relationships are general for different life-forms of C_3_ plants, species were categorized into the life-form groups of graminoids (grasses and sedges), forbs (herbaceous species other than graminoids), and shrubs. Multiple regressions were applied to determine if relationships between foliar elements and foliar δ^13^C remained significant after the altitudinal effect (combination of atmospheric pressure, precipitation, and thermal gradient effects) was accounted for. The data for multiple regressions were standardized in order to give equal weighting to the variables of N, P, K, and altitude.

## Results

### Correlations between foliar element and ratios

Correlations between site leaf elements contents and ratios showed that K, Ca, Mg and their sum were negatively correlated with C and were positively correlated with each other and with N and P. N and P were positively correlated but C was not correlated with either N or P. The C/N ratio showed negative correlations with P, K, Ca and Mg, and the C/P ratio with N, K, and Mg. The N/P ratio showed a weak positive correlation with C, a negative correlation with K and no correlation with Ca or Mg. ∑_K+ Ca+ Mg_ was negatively correlated with the C/N and C/P ratios but not with the N/P ratios (Table 1).

**Table 1:** Correlations (*r*) between foliar elements and ratios of C_3_ species sampled from 82 sites on the Qinghai-Tibet Plateau, China.

	N	P	K	Ca	Mg	C/N	C/P	N/P	∑_K+Ca+Mg_
C	0.041^ns^	−0.117^ns^	−0.184^**^	−0.267^***^	−0.268^***^	-	-	0.158^*^	−0.287^***^
N		0.517^***^	0.416^***^	0.355^***^	0.547^***^	-	−0.416^***^	-	0.510^***^
P			0.612^***^	0.246^***^	0.535^***^	−0.355^***^	−	-	0.565^***^
K				0.251^***^	0.493^***^	−0.378^***^	−0.243^***^	−0.432^***^	-
Ca					0.644^***^	−0.209^**^	−0.095^ns^	0.071^ns^	-
Mg						−0.416^***^	−0.397^***^	−0.101^ns^	-
C/N							-	-	−0.389^***^
C/P								-	−0.355^***^
N/P									−0.119^ns^

∑_K+ Ca+ Mg_, sum of the K, Ca and Mg concentrations in leaves. C/N, the ratio of foliar C concentration to N concentration; C/P, the ratio of foliar C concentration to P concentration; N/P, the ratio of foliar N concentration to P concentration.

“−”correlation rejected because of functional connection; ^***^, *P*<0.001; ^**^, *P*<0.01; ^*^, *P*<0.05; ns, not significant.

### Correlations between altitude and leaf element concentrations

**Figure 2:**
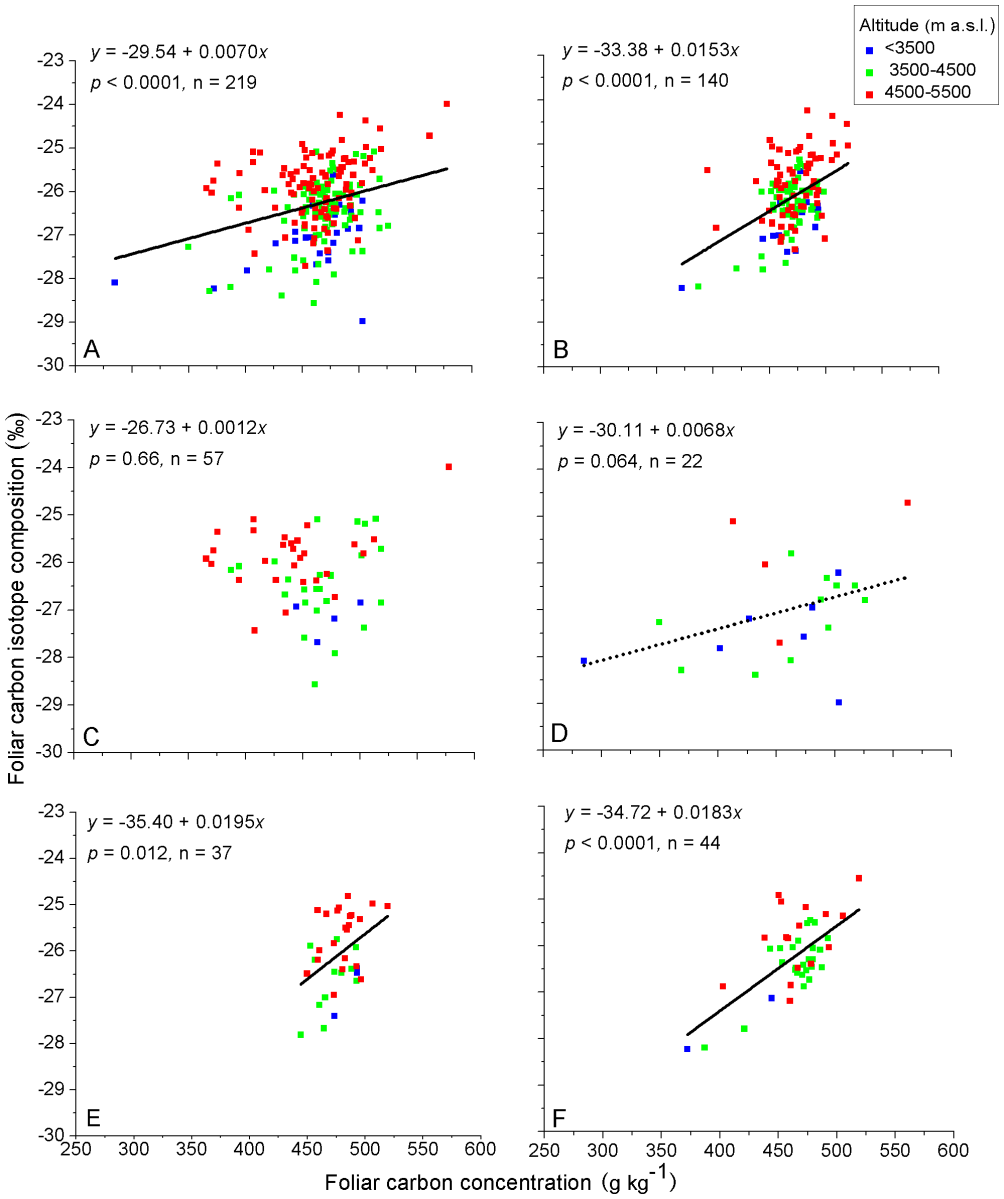
Relationships between foliar δ^13^C and foliar C of C_3_ plants on the Qinghai-Tibet Plateau, China. A) all samples, B) graminoids, C) forbs, D) shrubs, E) *Stipa* and F) *Kobresia*. Values for the linear regression (*y*) and significance (*P*) are shown for each relationship and the slope of the regression is plotted where it is significant. Solid line for significance at *P*<0.05, dashed line for significance at *P*<0.1. Sample points are color coded according to their location in three altitudinal ranges.

**Figure 3:**
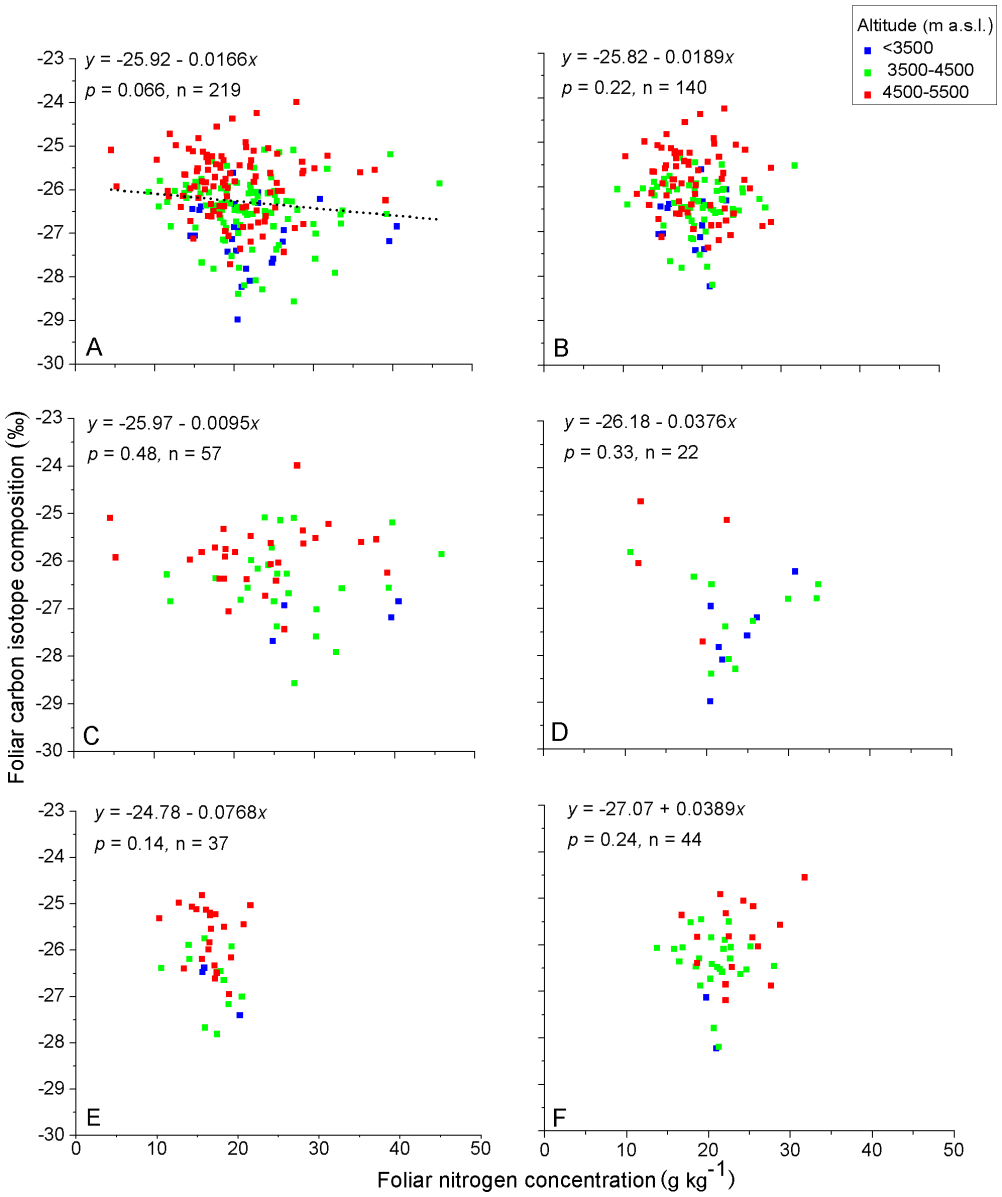
Relationships between foliar δ^13^C and foliar N of C_3_ plants on the Qinghai-Tibet Plateau, China. A) all samples, B) graminoids, C) forbs, D) shrubs, E) *Stipa* and F) *Kobresia*. Values for the linear regression (*y*) and significance (*P*) are shown for each relationship and the slope of the regression is plotted where it is significant. Solid line for significance at *P*<0.05, dashed line for significance at *P*<0.1. Sample points are color coded according to their location in three altitudinal ranges.

**Figure 4:**
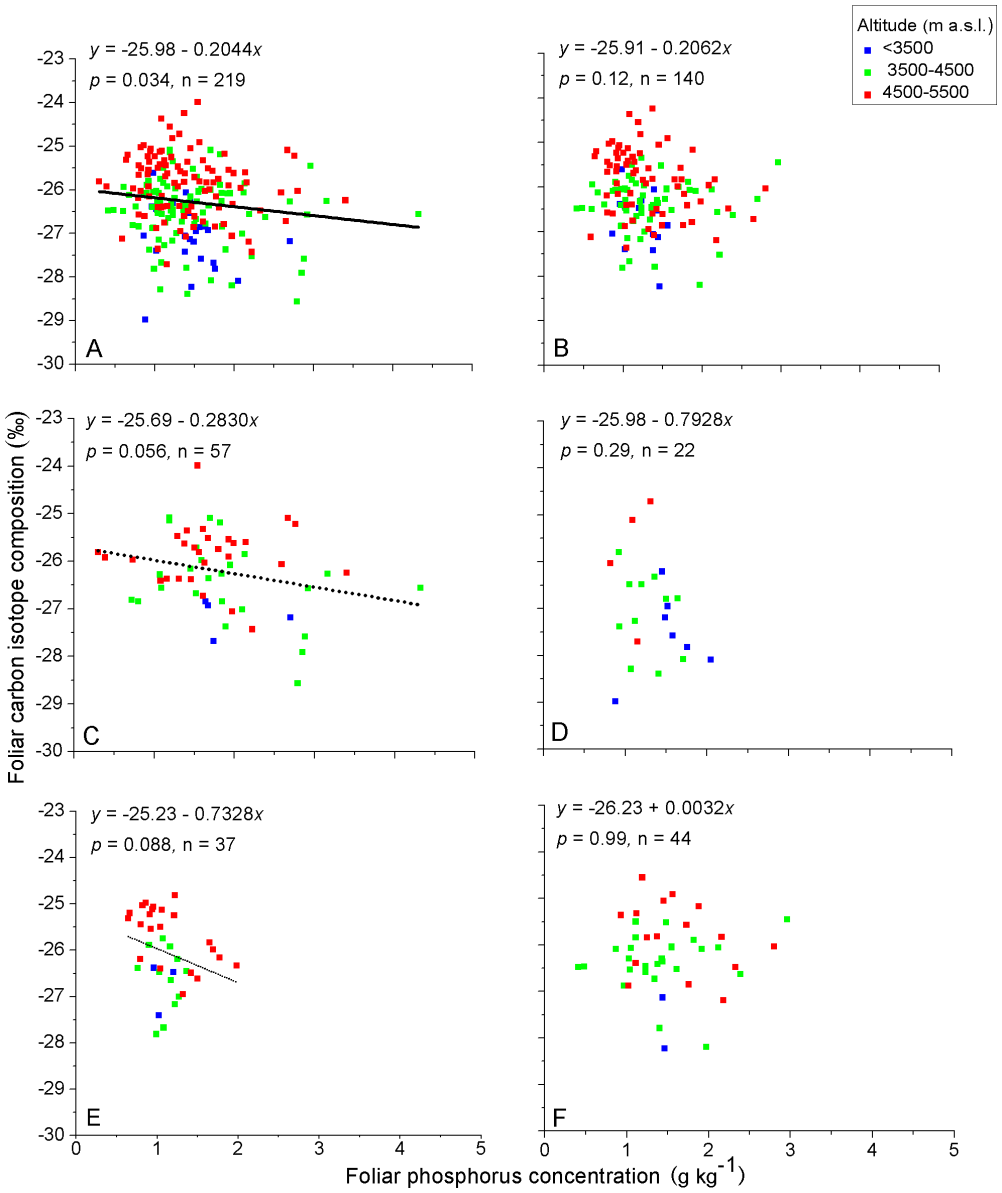
Relationships between foliar δ^13^C and foliar P of C_3_ plants on the Qinghai-Tibet Plateau, China. A) all samples, B) graminoids, C) forbs, D) shrubs, E) *Stipa* and F) *Kobresia*. Values for the linear regression (*y*) and significance (*P*) are shown for each relationship and the slope of the regression is plotted where it is significant. Solid line for significance at *P*<0.05, dashed line for significance at *P*<0.1. Sample points are color coded according to their location in three altitudinal ranges.

**Figure 5:**
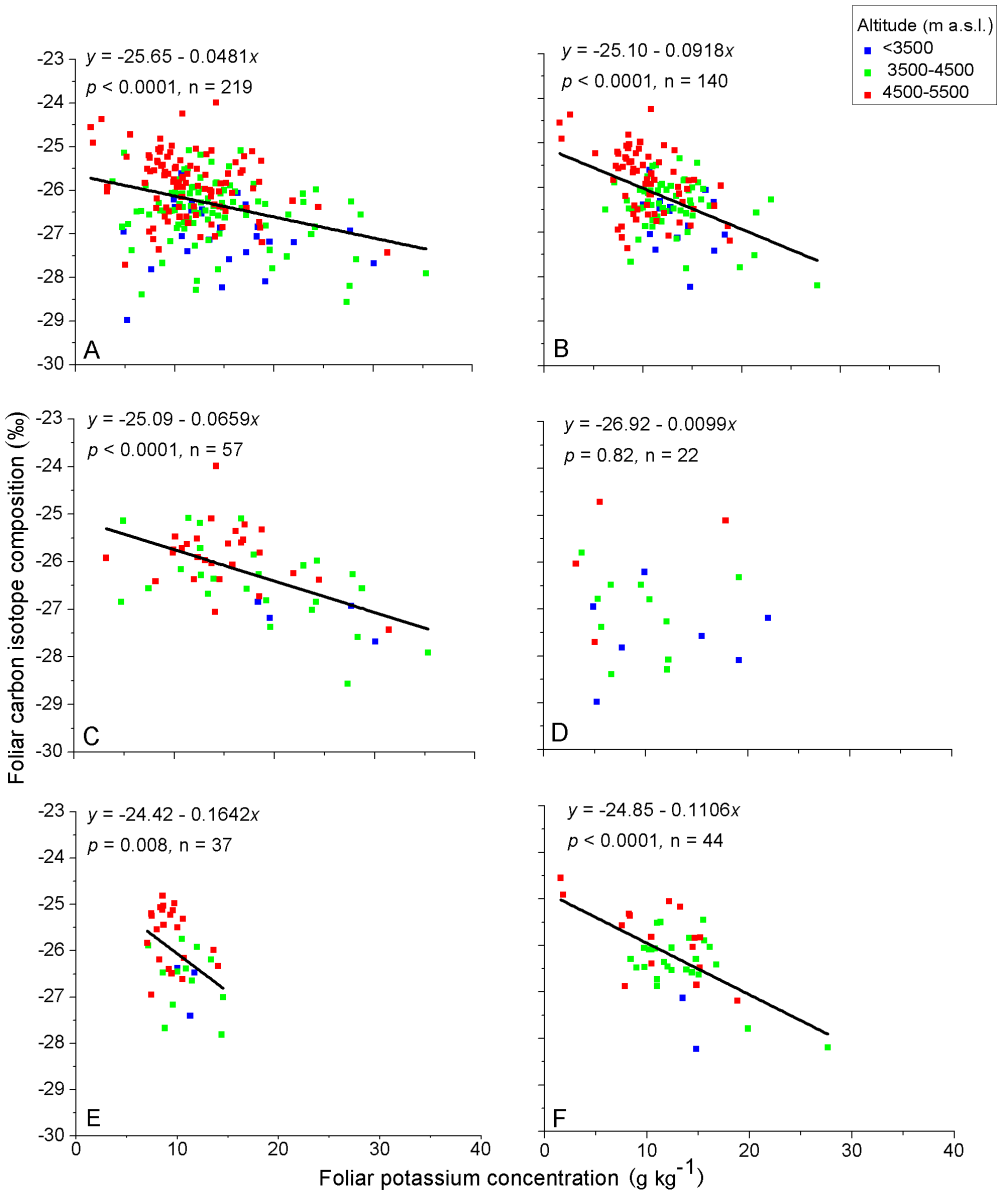
Relationships between foliar δ^13^C and foliar K of C_3_ plants on the Qinghai-Tibet Plateau, China. A) all samples, B) graminoids, C) forbs, D) shrubs, E) *Stipa* and F) *Kobresia*. Values for the linear regression (*y*) and significance (*P*) are shown for each relationship and the slope of the regression is plotted where it is significant. Solid line for significance at *P*<0.05, dashed line for significance at *P*<0.1. Sample points are color coded according to their location in three altitudinal ranges.

Altitudinal environmental gradients are the combination of atmospheric pressure, precipitation, and thermal gradient effects. Over the same set of sample sites, we found that foliar δ^13^C generally increased with altitude on the Qinghai-Tibet Plateau, China [7]. Investigating altitudinal patterns of foliar nutrient elements for taxonomic and life form groups of species (Table 2) in the context of foliar δ^13^C generally increasing with altitude may contribute towards better understanding of relationships between foliar δ^13^C and foliar nutrient elements. The color coding of the sample points for three ranges of altitude is given to assist visualization of the variation of element contents in relation to altitude (Figures 2, 3, 4, 5).

**Table 2:** Correlations (*r*) between foliar elements and altitude of taxonomic and life-form groups of C_3_ species sampled from 82 sites on the Qinghai-Tibet Plateau, China.

	C	N	P	K	Ca	Mg	C/N	C/P	N/P	∑_K+Ca+Mg_
All samples	−0.01^ns^	−0.22^**^	−0.04^ns^	−0.27***	0.10^ns^	−0.13^ns^	0.23***	0.09^ns^	−0.06^ns^	−0.12^ns^
*Kobresia*	0.39^**^	0.40^**^	0.19^ns^	−0.27^ns^	−0.02^ns^	0.05^ns^	−0.31^*^	−0.15^ns^	−0.04^ns^	−0.19^ns^
*Stipa*	0.14^ns^	−0.12^ns^	0.15^ns^	−0.49^**^	0.21^ns^	−0.16^ns^	0.10^ns^	−0.03^ns^	−0.11^ns^	−0.24^ns^
Graminoids	0.13^ns^	−0.04^ns^	0.10^ns^	−0.35***	0.22^**^	−0.10^ns^	0.11^ns^	−0.03^ns^	−0.08^ns^	−0.13^ns^
Forbs	−0.27^*^	−0.46***	−0.25^ns^	−0.46***	0.26^ns^	−0.18^ns^	0.37^**^	0.24^ns^	0.01^ns^	−0.24^ns^
Shrubs	0.03^ns^	−0.40^ns^	−0.57^**^	−0.34^ns^	−0.02^ns^	−0.59^**^	0.41^ns^	0.42^*^	0.03^ns^	−0.40^ns^

∑_K+ Ca+ Mg_, sum of the K, Ca and Mg concentration in leaves; C/N, the ratio of foliar C concentration to N concentration; C/P, the ratio of foliar C concentration to P concentration; N/P, the ratio of foliar N concentration to P concentration.

*** , *P*<0.001; ^**^, *P*<0.01; ^*^, *P*<0.05; ns, not significant.

The correlations between foliar C and altitude were significantly positive for *Kobresia* and negative for forbs. For all samples pooled, foliar N decreased with altitude, this was also the case for forbs, but foliar N increased with altitude for *Kobresia*. Shrubs showed a decline of foliar P with altitude, otherwise the correlations between P and altitude for other components were not significant. Foliar K decreased with altitude for all components although this correlation was not significant for *Kobresia* and shrubs. Except for the positive correlation of foliar Ca for graminoids and negative correlation of Mg for shrubs, Ca, Mg and ∑_K+Ca+Mg_ were not significantly correlated with altitude. Foliar C/N increased with altitude for all samples and forbs, but decreased for *Kobresia*. Except for shrubs which showed a positive correlation of C/P ratio with altitude, there were no significant correlations with altitude for foliar N/P and C/P (Table 2).

### Relationships between foliar δ^13^C and foliar C, N and P

For all samples pooled together, foliar C was positively related to foliar δ^13^C (Figure 2A). This relationship was more strongly defined for graminoids, *Stipa* and *Kobresia* (Figure 2B, E and F). The rates of increase of foliar δ^13^C relative to foliar C for *Stipa* and *Kobresia* were higher than that for all samples (*P* = 0.012). The relationship was marginally significant for shrubs (Figure 2D) and not significant for forbs (Figure 2C).

There were no significant relationships between foliar δ^13^C and foliar N regardless of life-form, genera or all samples together (Figure 3). For all samples pooled, foliar δ^13^C was negatively related to foliar P (Figure 4A). This trend was indicated for forbs (Figure 4C) but not for the other life form and taxonomic groups (Figure 4B, D, E, and F).

### Relationships between foliar δ^13^C and foliar mineral elements (foliar K, Ca, Mg and ∑_K+Ca+Mg_)

With the exception of shrubs, foliar K and ∑_K+Ca+Mg_ were negatively correlated with foliar δ^13^C. Significance of the correlation between foliar δ^13^C and foliar K was stronger than that between foliar δ^13^C and foliar ∑_K+Ca+Mg_. Negative correlations between foliar δ^13^C and both foliar Ca and Mg were indicated for graminoids but not for other species groups (Table 3). Regressions showing the relationships of foliar K with foliar δ^13^C for the species groups are plotted in Fig. 5.

**Table 3:** Correlations (*r*) of foliar δ^13^C with foliar mineral elements and foliar element ratios of leaf samples from 82 sites on the Qinghai-Tibet Plateau, China.

Species groups	Mineral elements	*R*	Element ratios	*r*
All samples	K	−0.32***	C/N	0.25***
	Ca	−0.06^ns^	N/P	0.03^ns^
	Mg	−0.10^ns^	C/P	0.15^*^
	∑_K+Ca+Mg_	−0.24***	-	-
Graminoids	K	−0.48***	C/N	0.24^**^
	Ca	−0.27^**^	N/P	0.03^ns^
	Mg	−0.20^*^	C/P	0.14^ns^
	∑_K+Ca+Mg_	−0.49***	-	-
Forbs	K	−0.56***	C/N	0.18^ns^
	Ca	0.21^ns^	N/P	0.17^ns^
	Mg	0.02^ns^	C/P	0.15^ns^
	∑_K+Ca+Mg_	−0.31^*^	-	-
Shrubs	K	−0.05^ns^	C/N	0.52^*^
	Ca	0.27^ns^	N/P	−0.11^ns^
	Mg	−0.27^ns^	C/P	0.29^ns^
	∑_K+Ca+Mg_	0.11^ns^	-	-
*Stipa*	K	−0.43^**^	C/N	0.30^ns^
	Ca	−0.05^ns^	N/P	0.21^ns^
	Mg	−0.32^ns^	C/P	0.40^*^
	∑_K+Ca+Mg_	−0.38^*^	-	-
*Kobresia*	K	−0.62***	C/N	−0.01^ns^
	Ca	−0.29^ns^	N/P	0.03^ns^
	Mg	−0.04^ns^	C/P	0.001^ns^
	∑_K+Ca+Mg_	−0.57***	-	-

∑_K+ Ca+ Mg_, sum of the K, Ca and Mg concentrations in leaves. C/N, the ratio of foliar C concentration to N concentration; C/P, the ratio of foliar C concentration to P concentration; N/P, the ratio of foliar N concentration to P concentration.

*** , *P*<0.001; ^**^, *P*<0.01; ^*^, *P*<0.05; ns, not significant.

### Relationships between foliar δ^13^C and foliar element ratios (foliar C/N, N/P and C/P)

For all samples pooled, foliar δ^13^C was positively related to foliar C/N and C/P, but the relationship between foliar δ^13^C and foliar N/P was not significant. Foliar δ^13^C was positively related to foliar C/N for graminoids and shrubs, but not with foliar N/P and C/P. Forbs had no significant relationships between foliar δ^13^C and the element ratios. *Stipa* had a significantly positive relationship between foliar δ^13^C and C/P, but otherwise the correlations for *Stipa* and *Kobresia* were not significant (Table 3).

### Relationships between foliar δ^13^C and atmospheric pressure, foliar N, P and K

After the altitudinal relationship with foliar δ^13^C was accounted for, of the foliar elements only K was significantly correlated to δ^13^C for all samples pooled. That was also the case for graminoids, forbs and *Kobresia*. However, K was not significantly related to δ^13^C for *Stipa* and none of the foliar elements were significantly related to δ^13^C for shrubs, possibly because the sample size for shrubs was small. Significantly, multiple regressions indicated that there were no correlation between foliar δ^13^C and foliar N irrespective of plant life form or taxonomic groups (Table 4).

**Table 4:** Multiple regressions between foliar δ^13^C (*y*) and altitude (*a.s.l.*), foliar N, foliar P and foliar K for all samples, graminoids, forbs, shrubs, *Kobresia* and *Stipa*.

Groups	a (constant)	b*x*_1_	Standardiz-ed coefficient (beta)	c*x*_2_	Standardiz-ed coefficient (beta)	d*x*_3_	Standardiz-ed coefficient (beta)	e*x*_4_	Standardiz-ed coefficient (beta)	*F*	*P*⋅multiple regressi-on
All samples (219)	−28.93***	3.69	0.45***	0.11	0.02^ns^	−0.07	−0.01^ns^	−1.07	−0.20^**^	21.45	***
Graminoids (140)	−27.38***	2.47	0.32***	−0.08	−0.02^ns^	0.17	0.04^ns^	−2.03	−0.38***	16.31	***
Forbs (57)	−28.29***	2.97	0.33^*^	1.22	0.27^ns^	−0.11	−0.02^ns^	−2.08	−0.50***	9.07	***
Shrubs (22)	−30.42***	4.45	0.51^ns^	−0.34	−0.06^ns^	0.10	0.02^ns^	0.57	0.13^ns^	1.40	ns
*Kobresia* (44)	−30.04***	5.11	0.49^**^	−0.004	−0.001^ns^	0.75	0.18^ns^	−2.08	−0.42^*^	6.99	***
*Stipa* (37)	−29.65***	5.56	0.68***	−0.40	−0.06^ns^	−1.96	−0.38^*^	0.28	0.05^ns^	8.31	***

The leaf samples were from 82 sites on the Qinghai-Tibet Plateau, China.

Numbers in brackets are the sample numbers for the groups. The regression model is *y* = a+b*x*_1_+c*x*_2_+d*x*_3_+e*x*_4_ where *y* is δ^13^C⋅(‰) and *x*_1_, *x*_2_, *x*_3_ and *x*_4_ are altitude, foliar N, foliar P and foliar K, respectively.

*** , *P*<0.001; ^**^, *P*<0.01; ^*^, *P*<0.05; ns, not significant.

## Discussion

### Relationships between altitude and leaf element concentrations

Considered for all samples foliar C content did not vary in relationship to altitude. While foliar C content of *Kobresia* increased and that of forbs decreased with altitude, it is not possible to explain the physiological or functional basis of these trends. Based on the assumption that the foliar element contents will reflect the availability of elements in the soil the plants grew in, the general conclusion could be made that the availability of soil N, and more consistently soil K, declined with altitude (Table 2). The same generalization cannot be made for P, Ca or Mg. However from variations of the relationships between altitude and element content for different taxonomic and life form categories it is apparent that factors other than the availability of elements in the soil influenced foliar element contents. The presence of the correlations with altitude raises the possibility of coincidental or spurious relationships of leaf element content to foliar δ^13^C arising from its well defined link to altitude.

### Relationships between foliar δ^13^C and foliar C, N and P

**Foliar C**. Previous research on foliar δ^13^C-foliar C concentration relationships is limited [38], but several studies have found foliar δ^13^C to be negatively related to ash content [22], [24]–[26], [41] due to passive accumulation of minerals in the vegetative parts of plants through the transpiration stream [42]. Generally, if ash content is higher, C content will be lower, and our result concur with this as foliar C content was negatively related to foliar K , Ca , Mg and ∑_k+Ca+Mg_ content (Table 1). Consequently there will be positive correlations between foliar δ^13^C and foliar C content (Figure 2A, E and F). However, the correlation between foliar δ^13^C and foliar C is not shown for forbs (Figure 2C). The reason for this is not known.

**Foliar N**. Our finding that there was no significant relationships between foliar δ^13^C and foliar N (Figure 3) agrees with the results found in high elevation areas by Ares and Fownes [43] and by Chen et al. [44]. However, correlations have been more frequently reported between foliar δ^13^C and leaf N concentration [28], [29], [32], [45]. These results usually build on the assumption that photosynthetic capacity increases with leaf N concentration. In our study for pooled samples, foliar N decreased with altitude (Table 2) a trend in agreement with Cordell et al. [46] who found that foliar N content as a proportion of biomass decreased by about 17% with increasing elevation. The usual trend is that foliar δ^13^C increases with altitude [4], [8] and this has been shown to be well defined for the region of this study [7]. This may be the reason for no positive relationship between foliar δ^13^C and leaf N (Figure 3A) and this is confirmed when the altitudinal effect is accounted for (Table 4). Even though, in contrast to the negative relationships for the other groupings of species, foliar N of *Kobresia* increased with altitude (Table 2), and there was no correlation between foliar δ^13^C and foliar N for this genus (Figure 3F). Thus another consideration, as indicated by Cordell et al. [36], is that photosynthetic capacity does not increase with increased leaf N concentration in areas of high elevation. Cordell et al. suggested that low temperatures and thicker leaves at high elevations may be partly responsible for nearly constant net assimilation rates across the altitudinal gradient despite the increase in foliar N with elevation. Thick leaves may allocate proportionally less N to photosynthesis than thin leaves, possibly by investing in N-containing secondary compounds for defense against predation or extreme environmental conditions [47]. Moreover, Reich et al [48] found that leaves with higher LMA (thicker leaves) frequently had a flatter slope for the relationship between leaf N and photosynthetic rate (mass-based). These findings show there is considerable variation in the response of fundamental leaf physiological/anatomical relationships to environmental variability, and highlight the need for establishing a rigorous environmental context for understanding such interactions [29].

**Foliar P**. Although leaf P is related to photosynthetic capacity through its effect on the enzyme Rubisco [49], research relating foliar P to foliar δ^13^C is limited. Merah [23] found no significant correlation between leaf P concentration and foliar δ^13^C, whereas we found a weak negative relationship between foliar δ^13^C and foliar P for all samples (Figure 4A), but not for the other groups of species. If this negative relationship is valid, a possible explanation is that the movement of P in the soil to the surfaces of roots where it can be absorbed into the plant partly depends on the mass flow of the soil solution resulting from transpiration by the plant [50], [51]. The finding that foliar P was positively related to foliar K, Ca, Mg and ∑_k+Ca+Mg_ (Table 1) supports this explanation.

### Relationship between foliar δ^13^C and foliar mineral elements

The negative correlations between foliar δ^13^C and foliar K and ∑_K+Ca+Mg_ (Table 3, Fig. 5) agree with the findings of several other studies [33], [42], [52]. These studies have postulated that K is passively accumulated in vegetative tissues by the transpiration stream and that plants that transpire more water per unit of dry matter produced (low δ^13^C and WUE) have higher concentrations of leaf K. However, other studies have shown positive correlation between foliar δ^13^C and foliar K [22], [31] and explain this by the key role K has in regulating stomatal movement.

We found that when the altitudinal effect was accounted for in multiple regressions (Table 4), there was significant negative relationship between foliar δ^13^C and foliar K for *Kobresia* but not for *Stipa* although there were significant negative relationships between foliar δ^13^C and foliar K for both *Kobresia* and *Stipa* in simple regressions (Fig. 5). A possible explanation is that as *Kobresia* generally occurs in wetter habitats, it is subjected to less water stress than *Stipa*, a genus typical of drier habitats. Consequently *Kobresia* can maintain a higher transpiration stream. Thus, the effect of foliar K on regulating stomatal movement is not important for *Kobresia* and K is accumulated in leaf by the transpiration stream leading to the negative correlation between foliar δ^13^C and foliar K. By comparison *Stipa*, in being more frequently subjected to water stress, displays a greater involvement of foliar K in the regulation of stomatal movement. This could offset the negative correlation between foliar δ^13^C and foliar K due to the accumulation of K in the leaf in the transpiration stream. This result suggests that the effect of foliar K on foliar δ^13^C is coincidental or spurious for *Stipa* due to foliar K of *Stipa* being significantly negatively correlated to altitude (Table 2).

Foliar K showed better correlation than foliar Ca and Mg with foliar δ^13^C, the correlations with Ca and Mg being significant only for graminoids (Table 3). This may be because foliar Ca and Mg concentrations are lower than that of foliar K, and antagonistic effects towards Ca and Mg by other cations (e.g., Na^+^, K^+^) counteract the transpiration effect on the accumulation of these cations in leaves. In addition, our results indicated that among the elements measured, K was the most important contributing element in the relationship between mineral content and foliar δ^13^C, a conclusion supported by Masle et al. [42].

### Relationship between foliar δ^13^C and foliar elemental ratios

With reference to the positive relationship between foliar δ^13^C and foliar C/N (Table 3), Li et al. [28] reported negative correlation between them and explained this on the basis that plants achieve higher water use efficiency (WUE) at the expense of decreased nitrogen use efficiency (NUE). However, Wittmer et al. [53] found that there was no relationship between foliar δ^13^C and foliar C/N. Our findings suggest that plants on the Qinghai-Tibet Plateau that have higher WUE may also have higher NUE, and on the basis of the significant correlation between δ^13^C and C/P for all samples and *Stipa* (Table 3), also have higher phosphorus use efficiency (PUE). This may be an adaptation of plants to the severe environmental conditions of the Plateau. The lack of correlation between foliar δ^13^C and foliar N/P (Table 3) is inconsistent with the finding of Cernusak et al. [50] that WUE (foliar δ^13^C) was related to foliar N/P. Cernusak et al. [37] suggested that foliar δ^13^C is positively related to foliar N/P according to the argument that plant carbon gain relates positively to the amount of foliar N and that the uptake of P partly depends on the mass flow of the soil solution resulting from transpiration by the plant [50], [51]. Consequently, it could be hypothesized that the N/P ratio would positively correlate with the C/T ratio, where T is the cumulative transpiration, and C/T is equal to WUE. However there may be some limitations when this hypothesis is applied to high elevation areas due to photosynthetic capacity remaining constant with variations of foliar N in these areas [36]. This could also account for the lack of correlation between foliar δ^13^C and foliar N/P on the Qinghai-Tibet Plateau.

## Conclusion

The study emphasizes the dominating influence of altitude related factors on variation of foliar δ^13^C and foliar elements. Consequently relationships between foliar elements and foliar δ^13^C that have been previously proposed appear to be secondary to other factors, and particularly we suggest atmospheric pressure and temperature, both of which decrease as altitude increases. Relationships between foliar N and δ^13^C expressed at low altitudes are suppressed at high altitudes. Relationships between foliar δ^13^C and K, possibly coincidental to transpiration flows, may be shown at high altitudes according to the water use efficiency of taxonomic or life-form groups considered.
